# Operations for Tuberculous Glands in the Neck

**Published:** 1910-06-11

**Authors:** 


### Surgery.

### OPERATIONS FOR TUBERCULOUS GLANDS IN THE NECK.

In a previous article dealing with tuberculous disease in lymphatic glands, we pointed out the apparently disproportionate frequency with which the cervical set of glands is attacked, and gave some explanations to account for this. In this article we propose to deal with the various operations for the relief of the condition. It will be taken for granted that medical treatment has been tried in all cases, and that in spite of this the glands have progressed in size; in other words, that a positive indication for operative interference exists.

### Technique of the Operation.

The technique of the operation must necessarily vary according to (i) the number of glands enlarged, and (ii) their exact pathological condition. It is, for instance, not at all uncommon to find a patient in whom some of the glands in each of the four subdivisions of the concatenate chain, previously mentioned, are enlarged. In such a condition a small straight incision over the most prominent part of the swelling is entirely inadequate to the needs of the case. For it will almost always be found that the enlargement which can be felt only represents quite a small proportion of the actual disease present. Further, in order to obtain a good result the whole chain of glands should, if possible, be removed *en masse*, and this can hardly ever be achieved through a linear incision, because the exposure afforded is inadequate. In these cases radical measures are demanded, and a large skin flap must be turned up, so that access may be obtained to both anterior and posterior triangles of the neck if need be. The best incision is a crescentic one, which starts below the lobule of the ear and is carried first backwards and downwards, almost skirting the margin of the hair and finally forwards to the origin of the sterno-mastoid muscle. This incision gives a very large exposure, and leaves a scar which is not at all obvious afterwards, since the greater portion of it is obscured by the patient's collar, and the part above this lies so far back as to be very little noticed.

The difficulty of enucleating the glands will vary in every case, but is, of course, much increased if they are matted together and densely adherent to the principal vessels of the neck. It is for this reason that it is advisable to define the lower limit of the mass and to start enucleating it from below upwards. If this is done the position of the main vessels, particularly the jugular vein, can be located, and the glands can be gradually stripped off it. But if the process is begun in a haphazard manner at an intermediate level, it is very easy to come down on the jugular vein, and possibly to damage or tear it, which is always an unpleasant *contretemps*, and may even lead to a catastrophe. Occasionally the glands are so closely connected with the vein that it may be necessary to remove it with the glands, but this is much less often called for in dealing with tuberculous than with malignant glands.

When the lower limit of the mass has been defined, the glands should be gradually dissected away from below upwards, and for this purpose a blunt instrument should always be used. The neck is a dangerous parish, and the point of a scalpel, however carefully used, is likely to do serious damage sooner or later. Sometimes a mass of glands in the anterior triangle is connected with a similar mass in the posterior underneath the sterno-mastoid. In such a case there should be no hesitation in dividing this muscle and uniting its cut ends subsequently. Before doing this the points of entrance and exit of the spinal accessory nerve in the muscle should be identified, and care should be taken to avoid injury to it. If the spinal accessory nerve is cut or damaged, wasting and paralysis of the corresponding trapezius muscle is liable to ensue, a result which causes a noticeable deformity. The closure of the wound is important. Some surgeons advocate the use of the subcuticular suture, but this is hardly feasible in the case of a long crescentic wound. Interrupted stitches of fine horsehair will generally give a good result, and will leave very little scarring if they are removed as soon as they have done their work. Some of the Margate surgeons, who have an unrivalled experience of this condition, remove them as early as the second day. It is well to put in a small drainage tube for twenty-four hours, since many lymphatic vessels are cut across and there is likely to be some oozing in the wound.

If only one of the four sets of the deep cervical glands appears to be infected, it is usually possible to remove them completely through a linear incision, which should be made, if possible, along one of the natural creases of the skin, so that the scar will subsequently show as little as possible. Thus, in removing the anterior group of the upper deep cervical glands an incision from the tip of the mastoid to the upper border of the thyroid cartilage will for this reason be found preferable to the incision parallel to the anterior border of the sterno-mastoid, which is usually advocated.

### The Golden Rule.

So far it has been supposed that the glands are not caseating, and that serious formation has not occurred. When this is the case it will usually be found that there are more glands, deep to the caseating one, in a less advanced pathological state, and a radical operation should always be performed if possible. It is best to make an elliptical incision, which includes the infected area of skin, and to attempt to dissect out the glands *en masse*. Generally, however, the abscess is accidentally opened in the course of these manipulations. If this occurs the pus should be wiped away and the cavity swabbed with carbolic acid to minimise the risk of infection, and the enucleation should then be proceeded with. These remarks apply equally to the presence of a sinus. The golden rule for obtaining a successful result in tuberculous glands in the neck is to do a sufficiently radical operation.

